# The impact of workplace violence, organizational climate, authentic leadership on job satisfaction among physicians in tertiary hospitals: the mediating role of job burnout

**DOI:** 10.1186/s12913-025-13453-7

**Published:** 2025-09-30

**Authors:** Wenjun Dai, Xuemei Zhang, Zhiping Liu, Ren Chen, Jing Cheng

**Affiliations:** 1 School of Health Management, Anhui Medical University, Hefei, Anhui 230032 China; 2 Sir Run Run Shaw Hospital, Zhejiang University School of Medicine, Hangzhou, Zhejiang 310016 China

**Keywords:** Job satisfaction, Job burnout, Authentic leadership, Organizational climate, Workplace violence

## Abstract

**Background:**

This study aims to investigate the direct associations among workplace violence, organizational climate, authentic leadership, and physicians’ job satisfaction, as well as the indirect pathways through job burnout, with the aim of offering potential preventive strategies.

**Methods:**

Through a purposive sampling method, a total of 408 questionnaires were distributed to physicians in six tertiary-level hospitals in Anhui Province, and 399 valid questionnaires were returned. Structural equation modeling was used to test the proposed model.

**Results:**

Although workplace violence did not directly influence physicians’ job satisfaction, it indirectly reduced it by exacerbating burnout. A positive organizational climate was shown to enhance job satisfaction directly and indirectly by mitigating job burnout. Authentic leadership directly enhanced job satisfaction and indirectly alleviated burnout by optimizing the organizational climate. Authentic leadership also indirectly enhanced job satisfaction by improving the organizational climate and alleviating job burnout.

**Conclusions:**

This study confirms that workplace violence, authentic leadership and organizational climate influence physicians’ job satisfaction through both direct and burnout-mediated indirect pathways. Interventions and programs promoting a positive work environment could potentially reduce job burnout and increase job satisfaction among Chinese physicians of tertiary hospitals.

## Background

Although job satisfaction of physicians has received substantial attention in Western countries since the 1990s [1], it is only in recent years that researchers or hospital managers in China have begun to address this issue [2]. Job satisfaction is defined as a positive emotional state resulting from the evaluation of one’s work or work experiences [3] and is recognized as a factor related to physician turnover and performance in the longer term [4, 5]. Furthermore, higher job satisfaction is associated with better quality of medical care [6], which in turn enhances patient adherence and patient satisfaction [7, 8]. However, investigations indicate that Chinese physicians report significantly lower job satisfaction compared to their Western counterparts [9–11] and professionals in other industries in China [12–14].

The first distinct challenge faced by physicians in tertiary hospitals is workplace violence(WPV), defined by the WHO as physical aggression (e.g., hitting, kicking, slapping, etc.) or psychological abuse (e.g., verbal abuse, threats, etc.) [15]. Media reports documented 345 hospital violent incidents in China between 2000 and 2020 [16], while a provincial survey revealed that 77.5% of physicians had experienced WPV, with 72.7% enduring psychological aggression [17]. The White Paper of Chinese Physicians also highlights that over half of physicians have encountered medical disputes or doctor-patient conflicts [18]. Such evidence points to the need for greater attention to the negative occupational conditions experienced by Chinese physicians [19].

The Job Demands-Resources (JD-R) model offers a comprehensive framework for examining factors influencing employees job satisfaction. It explains how job demands and resources predict psychological indicators and work experiences. According to the JD-R model, job demands including physical, social or organizational factors which require sustained effort, deplete energy and may lead to negative outcomes such as dissatisfaction. Building on this, Hockey’s compensatory control theory [20] posits that when job demands are high, individuals adopt performance-protection strategies involving sympathetic activation and/or increased subjective effort, which incur physical and psychological costs such as fatigue or exhaustion. As American clinical psychologist Freudenberger noted, job burnout occurs when work imposes excessive demands on an individual’s abilities, energy and resources, resulting in emotional exhaustion and depletion [21]. Given that WPV is a prominent issue in China’s healthcare system, its potential as a job demand for Chinese physicians warrants further exploration. Consequently, we hypothesized:

### Hypothesis 1

Higher physician-reported frequency of workplace violence would be directly related to more burnout(a), and less job satisfaction(b).

Affective Event Theory (AET) [22] complements the JD-R framework by emphasizing that work environments shape job satisfaction through employee’s emotional responses (e.g., burnout). Specifically, WPV as a negative work event, evokes emotional reactions that mediate the relationship between stressors and work experiences such as satisfaction [23]. A study on U.S. nurses showed that structural improvements like automated systems reduced burnout and dissatisfaction by alleviating emotional strain [24]. Additionally, WPV has been linked to turnover intentions through negative emotions such as fear [25]. Given that WPV’s role as a stressor which may trigger negative affect (AET) and ultimately reduce satisfaction, we hypothesized that:

### Hypothesis 2

Through the mediating effects of job burnout, workplace violence would be indirectly related to job satisfaction.

The second distinctive feature of Chinese physicians’ work context lies in the bureaucratic structure of public tertiary hospitals, characterized by rigid hierarchies, strict regulatory frameworks and rationalized, contract-based professional systems [26]. Concurrently, policy reforms such as tiered care and Diagnosis-Related Group (DRG) payment models have intensified administrative burdens and caseload complexity in these institutions. In summary, physicians are subject to dual management in both clinic practice and administrative tasks.

Against this backdrop, organizational climate, which refers to employees’ shared perception of daily policies, practices, procedures, and behavioral norms that are rewarded, supported, and expected within the organization [27], emerges as a critical contextual factor. Rooted in Gestalt psychology, this construct reflects a “whole” formed by interconnected experiences, capturing how physicians collectively perceive and interpretate their shared work environment [28]. In healthcare, organizational climate embodies domain-specific norms such as open communication, positive emotional dynamics, and fairness etc [29, 30]. These norms serve as critical cues that shape expectations around interpersonal reliability, information sharing, and equitable treatment. By embedding these norms in daily operations, it guides physicians’ perceptions of valued behaviors, directly influencing their workplace responses.

The link between organizational climate and job satisfaction is well established across various work groups, including university staff [31], kindergarten teachers [32] and nurses [33]. The present study aims to extend this knowledge to Chinese physicians, who are impacted by both hospital administration and system changes. Furthermore, evidence indicates that perceptions of a positive organizational climate are significantly associated with favorable mental health outcomes, such as lower levels of burnout, depression, and anxiety [34]. Existing literature also shows a bidirectional relationship between job burnout and job satisfaction [35, 36]. From an AET perspective, burnout can be categorized as an emotional response that ultimately influences job satisfaction. It is reasonable to assume that individuals’ perceptions of their work environment trigger emotional responses, which in turn shape their evaluations of work based on their beliefs and expectations. Thus we hypothesized:

### Hypothesis 3

Organizational climate would be directly related to job satisfaction(a), and indirectly related to job satisfaction through burnout(b).

Relational and task-based leadership are two distinct leadership styles. It is recognized that relational leadership predicts various positive outcomes for medical staff [37]. Authentic leadership, an emerging relational-oriented leadership style, is defined as a pattern of leader behavior rooted in self-awareness, balanced processing of information, relational transparency, and an internalized moral perspective. These behaviors encourage open sharing of information for decision-making and active acceptance of follower input [38]. Authentic leadership is suited to healthcare settings where ethical dilemmas, high-stakes decisions, and the need for trust are paramount [39–41]. By communicating positive emotions, hope, and trust, authentic leaders foster employees’ sense of responsibility and belonging, enhancing organizational commitment and eliciting better work attitudes and behaviors [42]. Avolio et al. further noted that authentic leaders promote higher quality relationships, encouraging active employee participation in the workplace, which in turn increases job satisfaction and performance [43]. A recent review systematically examined authentic leadership in healthcare, finding that it has both direct and indirect associations with a range of employee outcomes, including job satisfaction [44].

Leaders also play a critical in promoting positive organizational climate. Previous findings suggest that through relational transparency, an internalized moral perspective, and self-awareness, authentic leadership can facilitate a positive work climate by shaping the quality of support, information, and resources available in work areas [45]. Authentic leadership has been identified as an antecedent of organizational climate in the fields of business and nursing [43, 46].

Furthermore, as indicated by Blake et al. [47], authentic leadership promotes a positive organizational climate where everyone’s contributions are valued, trusted and respected. Working within a fair and supportive climate, job burnout which composed of emotional exhaustion and cynicism, is a rarely observed outcome. Idris et al. [48]. found that a positive organizational climate reduced emotional exhaustion in burnout assessment after three months. Ren [49] and Appelbaum [50] et al. also identified a positive role of organizational climate in mitigating job burnout. Evidence further shows that as job burnout increases, job satisfaction decreases [51]. As noted earlier, according to AET, perceptions of the work environment influence employees’ job attitudes by shaping their emotional experiences. While substantial research has examined the association between authentic leadership and outcomes in fields like business, authentic leadership has also been proposed as a fundamental component of effective leadership in healthcare, necessary for establishing healthy work environments and promoting positive outcomes for healthcare workers [44]. This study will provide empirical data to test these hypotheses by examining the direct effect of authentic leadership on job satisfaction and analyzing potential mechanisms in this relationship. Based on the literature and theoretical hypotheses discussed, we hypothesized the following:

### Hypothesis 4

Authentic leadership would be directly related to job satisfaction(a), and indirectly related to job burnout through organizational climate(b).

### Hypothesis 5

Through the mediating effects of organizational climate and job burnout, authentic leadership would be indirectly related to job satisfaction.

**Fig. 1:**
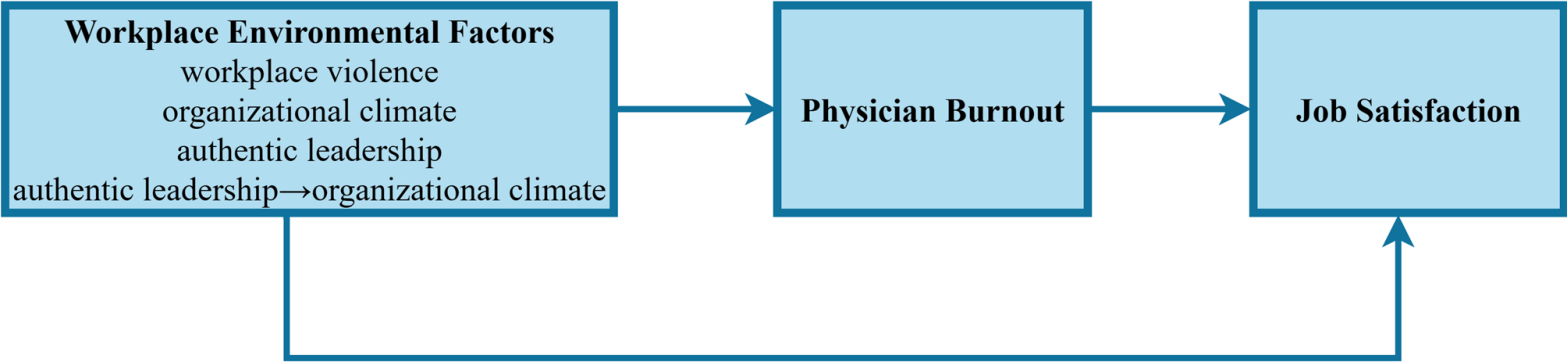
Theoretical model

To sum up, this study offers several contributions. First, by integrating the Job Demands-Resources (JD-R) model and Affective Event Theory (AET), it examines how workplace violence (a key job demand factor) and organizational climate/authentic leadership (critical job resources) influence job satisfaction through burnout (Fig. 1). This bridges fragmented healthcare research, as these factors have often been studied in isolation. Secondly, two separate structural equation models clarify the distinct mechanisms of negative and positive factors, avoiding ambiguity in complex models. Thirdly, the present study extends organizational climate research to Chinese physicians and adds to the understanding of how authentic leadership operates through climate to influence both burnout and job satisfaction, which may provide guidance for targeted interventions. Collectively, these contributions will enrich cross-cultural research on physicians’ attitudes and provide empirically grounded insights for targeted interventions aimed at improving job satisfaction among Chinese physicians, addressing both environmental challenges and supportive factors.

## Materials and methods

### Measures

#### Demographics and general information

Gender, age, educational level, marital status, professional title, administrative positions, years of practice and weekly work time (hours) of participating physicians were measured.

#### Workplace violence

The Chinese version of the Workplace Violence Scale was used to measure the frequency of workplace violence experienced by doctors from external sources (patients or their families) in the past 12 months. The scale has good reliability and validity among Chinese healthcare workers and consists of five items [52]. The first item concerns physical aggression (“In the past 12 months, have you been physically attacked, including being spit on, bitten, hit, or pushed”). The second item concerns emotional abuse, including being subjected to hurtful attitudes or remarks (such as insults, gestures, public humiliation, and coercion). The third item pertains to intimidation (such as verbal or written threats with the intent to harm). The fourth item assesses sexual harassment (such as repeated, unwanted intimate questions or remarks of a sexual nature). The fifth item pertains to sexual aggression, which includes any forced physical sexual contact. Each item was scored on a 4-point scale reflecting the respondent’s frequency of exposure to such violence (0 = never, 1 = once, 2 = twice to three times, and 3 = more than four times). The total possible score ranges from 0 to 15, with higher scores indicating greater frequency of exposure to workplace violence.

#### Authentic leadership

The Chinese version of the Authentic Leadership Inventory [53] was used to assess physicians’ perceptions of their immediate supervisors’ leadership styles. This inventory consists of 14 items categorized into four dimensions: three items on self-awareness (i.e., demonstrating an understanding of one’s strengths and weaknesses and the multifaceted nature of the self; e.g., item 4: ‘My leader solicits feedback to improve his or her interactions with others’); three items on relationship transparency (e.g., item 1: ‘My leader clearly states what he means’); four items on moral-ethical perspective (i.e., acting on one’s internal moral standards and values; e.g., item 2: ‘My leader demonstrates consistency between his beliefs and actions’); and four items on balanced processing (i.e., objectively analyzing all relevant data before making a decision; e.g., item 3: ‘My leader asks for ideas that challenge his core beliefs’). Responses were made on a Likert scale ranging from 1 “strongly disagree” to 5 “strongly agree”. The dimension score is calculated by adding up the scores for each dimension, and the total score (range:14–70) reflects the doctor’s perception of authentic leadership. The higher the score, the higher the doctor’s perception of authentic leadership.

#### Organizational climate

Organizational climate, which reflects the employees’ perception of the organizational culture and is easily measured through employee questionnaires [54]. Considering the cultural variance, the organizational climate scale developed by Chinese researchers [55] was adopted. The scale consists of 12 items and can be divided into 4 four dimensions: 3 items of organizational trust climate (e.g., item 1: ‘My colleagues are trustworthy’), 3 items of organizational communication climate (e.g., item 5: ‘Colleagues often exchange ideas’), 3 items of organizational emotional climate (e.g., item 8: ‘At work, colleagues are optimistic and confident’), and 3 items of organizational fairness climate (e.g., item 11: ‘In an organization, employees who make great contributions get great rewards’). All items were measured on 5-point scales ranging from 1 “strongly disagree” to 5 “strongly agree”. The score for each dimension is calculated as the sum of its respective items, while the total score (range: 12–60) is the sum of all 12 items. This total score reflects doctors’ perceptions of organizational climate, with higher scores indicating more positive perceptions. The questionnaire was distributed to each physician to elicit everyone’s perceived organizational climate.

#### Job burnout

This study used the Chinese version of the Maslach Burnout Inventory-General Services which was developed by Maslach and Jackson [56], then cross-culturally adapted for Chinese populations by Professors Li and Shi of the Chinese Academy of Sciences [57], and published in the Chinese Journal of Psychology [58], is a valid tool for assessing job burnout in the Chinese population. The 15-item assessment evaluates and scores the 3 dimensions of job burnout: emotional exhaustion (5 items) covers the experience of both emotional and physical exhaustion; cynicism (4 items) reflects indifference, detachment from work and active disengagement from work; professional efficacy (6 items) consists of feelings of competence, achievement and accomplishment in one’s work. Each item consists of a 7-point Likert scale ranging from 0 (“never”) to 6 (“daily”), and the 6 professional efficacy items need to be reverse-scored. Dimension scores are calculated by summing respective items, while the total score (range: 0–90) reflects overall burnout severity, with higher scores indicating greater burnout.

#### Job satisfaction

The Chinese version of the Michigan Organization Assessment Questionnaire Work Satisfaction Subscale [59] was adopted. The questionnaire includes three items: ‘Overall, I like working here’; ‘Overall, I do not like my job’ (reverse-scored); and ‘Overall, I am satisfied with my job.’ Responses were rated on a 5-point Likert scale, with scores ranging from 1 ‘Strongly disagree’ to 5 ‘Strongly agree,’ yielding a total score range of 3 to 15 points, with higher total scores indicating higher job satisfaction. This scale is widely used in international research and is considered a reliable and valid tool for measuring job satisfaction.

### Study subjects and procedures

A cross-sectional study of physicians was conducted from September to December 2021 in Anhui Province, China. The purposive sampling method was used to select 6 tertiary class A hospitals (≥ 500 beds) in the whole of Anhui province, which have a comparable capacity of medical and health care services. Since the JD-R model explore the job demand or resource factors for a relatively long time [60], whereas the AET theory examines the impact of emotional events on stable occupational groups [61]. Therefore the inclusion criteria for physicians were (1) at least 1 year of clinical experience in the current hospital, (2) still in clinical practice during the study, and (3) voluntary participation in this study.

After receiving permission from each hospital, researchers who had undergone standardized training conducted face-to-face surveys using short anonymous questionnaires. The questionnaires were distributed on site, the purpose of the study was explained, and the questionnaires were collected immediately. A total of 408 questionnaires were distributed to physicians, and 399 valid questionnaires were returned (response rate = 97.8%). The departments that were surveyed included surgery, internal medicine, obstetrics and gynecology, pediatrics, ophthalmology, and emergency medicine in the 6 hospitals.

The study was approved by the Ethical Committee of Anhui Medical University (Grant Number 83220199).

### Demographic characteristics of participants

A total of 399 physicians participated in this study and their demographic profile is presented in Table 1.

**Table 1:** Demographic characteristics of participants (*n* = 399)

Variables	*n* (%)	Variables	*n* (%)
Gender		Marital status	
Men	230(57.6)	Married	329(82.5)
Women	169(42.4)	Single/divorced/widowed	70(17.5)
Age group(years)		Administrative positions	
≤ 30	85(21.3)	Yes	39(9.8)
31 ~ 50	295(73.9)	No	360(90.2)
≥ 51	19(4.8)	Years of practice	
Educational level		≤ 5	114(28.6)
Bachelor’s degree	134(33.6)	6 ~ 10	113(28.3)
Master’s degree	157(39.3)	11 ~ 20	117(29.3)
Doctorate degree	108(27.1)	≥ 21	55(13.8)
Professional title		Weekly work time (hours)	
Primary title	123(30.8)	<40	93(23.3)
Intermediate title	167(41.9)	≥ 40	306(76.7)
Senior title	109(27.3)		

### Analytical plan

SPSS 26.0 and AMOS 24.0 software was applied. In the descriptive analysis, measurement data were expressed as means and standard deviations, while count data were expressed as frequencies or percentages. Cronbach’s α coefficient method was used to check the reliability of the scales with an acceptable value of ≥ 0.6 [62, 63]. Pearson’s correlations were used to examine correlations between the continuous variables.

Two structural equation models were constructed to analyze the path relationship between work environment and job satisfaction. Taking workplace violence, job burnout and job satisfaction as potential variables, and each indicator scale as observation variables, model 1 was constructed. Taking authentic leadership, organizational climate, job burnout and job satisfaction as potential variables, and each indicator scale as observation variables, model 2 was established. The steps involved in the AMOS were as follows: construct the model–import relevant data–set the model parameters–evaluate the model–modify the indicators–obtain the best model. In this study, the evaluation indices of the model fit index are as follows: Chi-square/degrees of freedom ratio (CMIN/DF) < 5, comparative fit index (CFI) > 0.90, root mean square error of approximation (RMSEA) < 0.08, tucker-lewis index (TLI) > 0.90, and incremental fit index (IFI) > 0.90 [64–66]. The mediation effect test was performed using Bootstrap 1000 samples. The significance level was defined as a p-value of < 0.05 for all analyses.

## Results

### Common method bias test

Since all the data collected in this study were self-reported, it is necessary to conduct a common method bias test before proceeding with further analysis and processing. This study used the Harman single-factor test to examine whether all items related to the study variables exhibited common method bias. The Harman single-factor test is a statistical method that uses exploratory factor analysis to detect common method bias. In cross-sectional studies if the proportion of variance explained by a single factor exceeds 40%, it is considered that there is significant common method bias [67]. The results of exploratory factor analysis showed that the first factor explained 34.54% of the total variation and less than the 40% critical threshold. Therefore, no serious problem of common method bias was found in the current study.

### Assessment of the instrument reliability and validity

As shown in Table 2, the absolute values of skewness ranged from 0.074 to 1.633 (all < 2), and the absolute values of kurtosis ranged from 0.114 to 2.630 (all < 7), meeting the criteria for normal distribution. The Cronbach’s alpha coefficients for each scale ranged from 0.632 to 0.963, indicating good internal consistency; the CR values were all greater than 0.7, meeting the recommended standards by Hair et al. [68]; the AVE values were all above 0.5, demonstrating good convergent validity [69]. In summary, all scales exhibited acceptable reliability and validity.

**Table 2:** Reliability and validity test of the instruments

Variables	Skew	Kurt	α	CR	AVE
JB	-0.137	-0.166	0.888	0.965	0.648
WPV	1.633	2.630	0.632	0.884	0.610
AL	-0.226	0.114	0.963	0.971	0.696
JS	-0.074	0.368	0.818	0.898	0.748
OC	0.127	-0.141	0.960	0.946	0.599

α Cronbach’s alpha; CR composite reliability; AVE average variance extracted; JB job burnout; WPV workplace violence; AL authentic leadership; JS job satisfaction; OC organizational climate

### Descriptive statistics and correlations between variables

Approximately 54.4% of the participants reported at least one type of workplace violence occurring in the past 12 months. Emotional abuse accounted for the majority of workplace violence (50.4%), followed by threats of intimidation (21.8%) and physical aggression (11%).

Before testing the model, we generated a correlation matrix as shown in Table 3. The results showed that the respondents’ workplace violence scores were 1.45 ± 1.90 (out of 15 points), authentic leadership scores were 50.54 ± 9.99 (out of 70 points), organizational climate scores were 42.22 ± 8.49 (out of 60 points), job burnout scores were 35.22 ± 12.14 (out of 90 points), and job satisfaction scores of 11.20 ± 2.02 (out of 15). The results demonstrated that higher levels of workplace violence correlated with increased job burnout (*r* = 0.296, *p* < 0.001) and decreased job satisfaction (*r*=-0.253, *p* < 0.001). Authentic leadership was positively correlated with both organizational climate (*r* = 0.619, *p* < 0.001) and job satisfaction (*r* = 0.388, *p* < 0.001), but negatively correlated with job burnout (*r*=-0.293, *p* < 0.001). In addition, organizational climate was negatively correlated with job burnout (*r*=-0.383, *p* < 0.001) and positively correlated with job satisfaction (*r* = 0.435, *p* < 0.001).

**Table 3:** Descriptive statistics and the correlation coefficients of the variables

Variables	M ± SD	WPV	AL	OC	JB	JS
WPV	1.45 ± 1.90	1				
AL	50.54 ± 9.99	-0.135^**^	1			
OC	42.22 ± 8.49	-0.253^**^	0.619^**^	1		
JB	35.22 ± 12.14	0.296^**^	-0.293^**^	-0.383^**^	1	
JS	11.20 ± 2.02	-0.253^**^	0.388^**^	0.435^**^	-0.627^**^	1

** P < 0.001

### Structural model

**Fig. 2:**
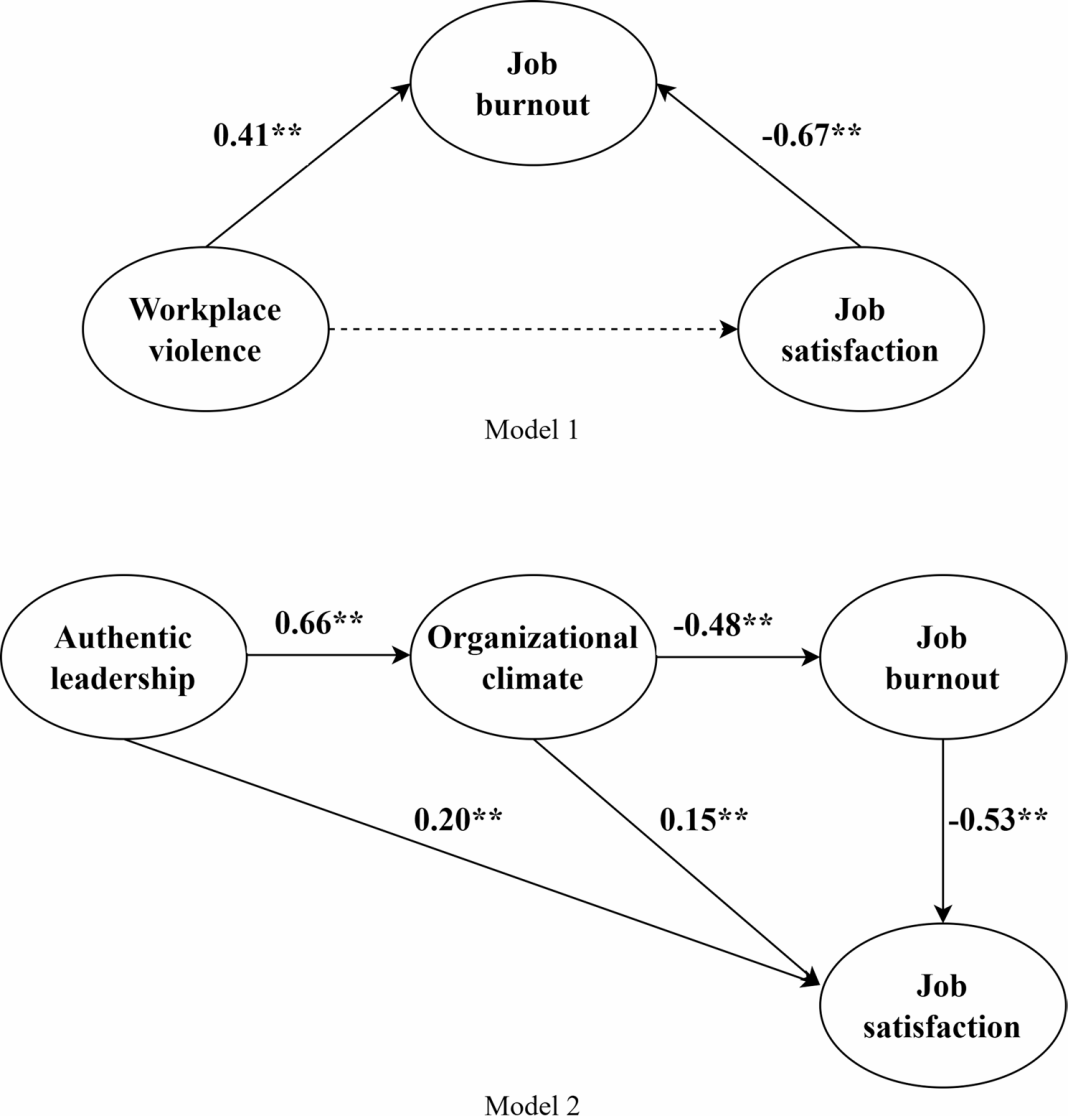
The final structural equation model

The initial model fitting results showed that some fitting indices did not meet the ideal fit values. Therefore, the model was revised by establishing relationships between the residuals, as shown in Fig. 2, to obtain the final structural equation model. The model fitting results showed satisfactory values in Table 4, with all fitting indices meeting the standards, indicating good overall fit. As presented in Table 5, workplace violence had a significant direct effect on job burnout (β = 0.413, *p* < 0.001), which supported H1a. A good organizational climate led to high job satisfaction among physicians (β = 0.150, *p* = 0.021), supporting H3a. Authentic leadership significantly and directly influenced job satisfaction (β = 0.195, *p* < 0.001), supporting H4a. Additionally, a positive organizational climate effectively reduced the degree of job burnout (β=-0.478, *p* < 0.001), and reduced job burnout enhanced job satisfaction (β=-0.527, *p* < 0.001). Authentic leadership played a significant promotional role in shaping the organizational climate (β = 0.657, *p* < 0.001).

**Table 4:** Goodness-of-fit indexes of models

Goodness criteria of model fit	CMIN/Df^a^	CFI^b^	RMSEA^c^	TLI^d^	IFI^e^
Model 1	3.334	0.942	0.077	0.915	0.942
Model 2	2.476	0.978	0.061	0.972	0.979
Acceptable level	< 5	> 0.9	< 0.08	> 0.9	> 0.9

^a^ Chi-square/DF, ^b^ Comparative Fit Index, ^c^ Root Mean Square Error of Approximation, ^d^ Tucker-Lewis Index, and ^e^ Incremental fit index

**Table 5:** The test of path coefficient by MLE

Path	Standardized coefficient β	Non-standardized coefficient			Result
		S.E	C.*R*	*P*	
WPV → JB	0.413	2.639	5.185	<0.001	Supported
WPV → JS	0.001	0.287	0.020	0.984	Not supported
OC → JB	-0.478	0.155	-7.813	<0.001	Supported
OC → JS	0.150	0.025	2.314	0.021	Supported
JB → JS	-0.527	0.009	-8.971	<0.001	Supported
AL → OC	0.657	0.040	12.881	<0.001	Supported
AL → JS	0.195	0.017	3.447	<0.001	Supported

The statistical significance of the mediating effect was tested using the bootstrap method [70]. As shown in Table 6, the indirect effect of workplace violence on job satisfaction through job burnout is -0.274, with a 95% confidence interval of -0.375 to -0.199. Since this confidence interval does not include 0, it supports H2. Similarly, the indirect effect of organizational climate on job satisfaction through job burnout is 0.252, with a 95% confidence interval of 0.178 to 0.338, supporting H3b. The indirect effect of authentic leadership on job burnout through organizational climate is -0.314, with a 95% confidence interval of -0.402 to -0.239, supporting H4b. Authentic leadership→Organizational climate→Job burnout→Job satisfaction, with an indirect effect value of 0.165 and a corresponding confidence interval of 0.114 to 0.233, supporting H5.

**Table 6:** Coefficients of the structural equation mediation model

	Estimate	Boot strap SE	Bootstrap 95% CI	
			Lower	Upper
Direct effects				
WPV → JS	0.001	0.078	-0.147	0.144
OC → JS	0.150	0.086	-0.013	0.319
AL → JB	0.000	0.000	0.000	0.000
AL → JS	0.195	0.073	0.040	0.332
Indirect effects				
WPV → JB → JS	-0.274	0.044	-0.375	-0.199
OC → JB → JS	0.252	0.040	0.178	0.338
AL → OC → JB	-0.314	0.040	-0.402	-0.239
AL → OC → JS	0.098	0.057	-0.007	0.215
AL → OC → JB → JS	0.165	0.030	0.114	0.233
Total effects				
WPV → JS	-0.273	0.066	-0.401	-0.145
OC → JS	0.402	0.076	0.252	0.560
AL → JB	-0.314	0.040	-0.402	-0.239
AL → JS	0.459	0.056	0.355	0.569

## Discussion

### Principal findings and practical implications

This study demonstrates that workplace violence (WPV) against Chinese physicians is prevalent, with 54.4% of respondents reporting at least one incident in the past 12 months. This finding aligns with previous research, confirming that violence against physicians remains a pressing social issue in China [71]. Furthermore, the present study validates that WPV constitutes a critical job demand for Chinese physicians. While WPV showed no direct effect on job satisfaction, it significantly increased job burnout, which in turn reduced job satisfaction. According to the JD-R model, high-stress work environments (such as those affected by WPV) require individuals to invest additional energy in coping and goal achievement, leading to the depletion of physical and psychological resources. WPV in healthcare settings not only threatens physicians’ personal safety and mental health but also triggers resource depletion and emotional exhaustion. In severe cases, experiences of disrespect from patients or safety fears may even lead physicians to question their professional value. These processes collectively contribute to elevated job burnout, which our findings confirm is negatively associated with job satisfaction.

This pathway whereby WPV impacts job satisfaction through job burnout, offers several practical implications. First, targeted interventions to mitigate WPV are imperative. Current strategies prioritize individual-level education, including teaching patients respect and training physicians in patient-centered care. This ignores systemic variables, such as excessive outpatient visits, which limit the time available for communication between doctors and patients. Therefore, the solution to WPV must also address the distribution of healthcare resources and strengthen legal protection for physicians. Secondly, since burnout predicts job satisfaction as an antecedent variable, hospital managers can monitor physicians psychological states (e.g., job burnout) to provide early warnings for potential declines in employee’s job satisfaction. This allows timely intervention to maintain satisfaction levels, which is a nationally monitored indicator for public tertiary hospitals [72].

The findings also show a dual impact of organizational climate on physicians’ job satisfaction: it enhances satisfaction through a direct pathway and exerts an indirect positive effect by reducing job burnout. These results are consistent with previous domestic studies, which have implied that positive work resources such as good colleague relationships and organizational support are correlated with reduced negative emotions (e.g., job burnout) and positive work attitudes (e.g., job satisfaction) [73, 74]. Meanwhile, the present study further validates the assumption that organizational climate is an important work resource for physicians. Organizational climate compensates for the resource depletion that physicians experience when coping with job demands. The AET further supplements this logic that individuals’ perception of positive work environment triggers emotional reactions, which in turn influence subsequent attitudes and behaviors. Qi et al. [75]. have observed in their study on nurses that an unfriendly work environment exacerbates emotional exhaustion and negatively affects work attitudes. This study verifies the role of a positive environment in the physician group. Physicians’ positive perception of organizational climate reduces job burnout (negative emotions), and the reduction in burnout further improves job satisfaction. This finding highlights the critical role of emotional experience in the formation of job satisfaction.

Since the multidimensional components of organizational climate (trust, communication, fairness, etc.) collectively contribute to aforementioned effects, they provide specific elements that help us to understand the overall work climate. As the foundation of organizational operations, trust can enhance the sense of belonging [76] and reduce job burnout caused by uncertainty in interpersonal relationships. In collaborative medical settings, open and smooth communication can improve psychosomatic well-being [77], reduce interpersonal conflicts [78], and directly promote satisfaction. Additionally, distributive and interactional fairness enables physicians to perceive “equity between effort and reward,” thereby enhancing work engagement and reducing job burnout. These mechanisms are consistent with Schneider’s view that “organizational climate directly affects attitudes by conveying supportive signals“ [79] and also confirm its indirect role in protecting psychological resources and reducing job burnout.

In summary, the results of this study suggest that creating such a positive organizational climate can significantly reduce job burnout and improve satisfaction with their jobs. These findings provide practical implications for healthcare institutions. Medical institutions can foster collaboration and communication between supervisors, subordinates and colleagues by organizing activities and establishing unobstructed communication channels, thereby creating an organizational climate of mutual trust and effective communication. At the same time, they should pay attention to the emotional well-being of physicians within the organization and strive to foster a culture of collective vitality, optimism and solidarity. They should also establish fair and just performance evaluation and promotion mechanisms.

Research on leadership has yielded extensive findings in foreign nursing populations [44, 80], with multiple studies confirming the associations between authentic leadership and nurses’ job satisfaction [46, 81] as well as burnout [82]. However, in the Chinese healthcare context, medical professionals, particularly physicians, are primarily emphasized for their professional expertise, while their role within the management systems of healthcare organizations is overlooked. To our knowledge, no studies have examined how leadership factors influence Chinese physicians’ mental health or job satisfaction. Our research addresses this gap by demonstrating that authentic leadership not only directly enhances physicians’ job satisfaction but also exerts indirect positive effects by improving organizational climate and reducing job burnout. These findings underscore the urgency of strengthening leadership practices in Chinese public hospitals.

Previous research has suggested that leadership connects to job satisfaction through empowering subordinates [83] and alleviating compassion fatigue [84]. This study introduces physicians perceived organizational climate as an explanatory mechanism. Authentic leaders demonstrate core values through transparent communication, objective information sharing, and consistent behaviors, thereby fostering a supportive organizational climate. These behaviors establish normative standards that are internalized by followers, reinforcing shared values across the organization. In such a climate characterized by trust and effective communication, physicians tend to report higher work-related self-efficacy and optimism, which in turn reduces job burnout and enhances job satisfaction.

Based on these findings, healthcare institutions should prioritize authentic leadership capabilities when selecting clinical department directors and managers, as well as in their performance evaluations. Structured development programs, including targeted training and mentoring initiatives, should be implemented to strengthen authentic leadership among medical leaders.

### Limitations and future research directions

There are several limitations to this study that must be noted. Firstly, the cross-sectional design restricts our ability to observe dynamic changes between the exploratory variables and the outcomes over time, thus hindering causal inferences. Secondly, the influence of covariates was not considered in the structural equation model (SEM). Participants’ demographic factors and other individual-level factors related to job characteristics (e.g. weekly working hours) may also affect the variables in this study.

Despite these limitations, the preliminary findings of this study provide insights for future research. First, this study initially verified the effects of the total score of organizational climate and authentic leadership on physicians’ psychological states and work attitudes. Future research should further analyze the magnitude and pathways of influence of each sub-dimension of the two variables on employees’ outcomes. For instance, within organizational climate, it is necessary to determine whether fairness climate or other specific sub-dimensions (e.g., communication climate, emotional climate) exert a stronger positive effect, which would help prioritize intervention strategies. Second, this study collected data on organizational climate using personal perceptions at the individual level. Future studies could integrate individual- and organizational-level data to conduct cross-level analyses, thereby exploring the cross-level transmission mechanism of organizational climate in shaping physicians’ psychological states and work attitudes. Third, with the implementation of healthcare reform policies such as balanced resource allocation, continuous attention should be paid to the trends of workplace violence and its impacts on physicians’ physical and mental health at work. Multilevel strategies including organizational policies, training programs, and psychological support systems should be developed to mitigate its occurrence and harm.

## Conclusions

The present study demonstrates that multidimensional environmental factors from both job demands and resources, specifically workplace violence, organizational climate, and authentic leadership, exert significant direct or indirect influences on satisfaction pathways among Chinese physicians, which constitute notable omissions in extant Chinese healthcare literature. This evidence necessitates re-conceptualization of physician well-being through dual lenses as mitigating negative environmental factors while strategically cultivating positive dimensions. Consequently, healthcare administrators should prioritize evidence-based interventions that simultaneously address environmental threats and foster supportive hospital administration ecosystems.

## Data Availability

The datasets generated and/or analyzed during the current study are not publicly available but are available from the corresponding author on reasonable request.

## References

[1] Konrad TR, Williams ES, Linzer M, et al. Measuring physician job satisfaction in a changing workplace and a challenging environment. SGIM career satisfaction study group. Society of general internal medicine. Med Care. 1999;37(11):1174–82. 10549620 10.1097/00005650-199911000-00010

[2] Chen C, Ding X, Li J. Transformational leadership and employee job satisfaction: the mediating role of employee relations climate and the moderating role of subordinate gender. Int J Environ Res Public Health. 2021;19(1):233. 35010493 10.3390/ijerph19010233 PMC8744760

[3] Locke EA. The nature and causes of job satisfaction. In: Dunnette MD, editor. Handbook of industrial and organizational psychology. Chicago, IL: Rand McNally; 1976. pp. 1297–349.

[4] Ran L, Chen X, Peng S, Zheng F, Tan X, Duan R. Job burnout and turnover intention among Chinese primary healthcare staff: the mediating effect of satisfaction. BMJ Open. 2020;10(10):e036702. 33033013 10.1136/bmjopen-2019-036702 PMC7542935

[5] Liu D, Yang X, Zhang C, et al. Impact of job satisfaction and social support on job performance among primary care providers in Northeast China: a cross-sectional study. Front Public Health. 2022;10:884955. 35801248 10.3389/fpubh.2022.884955 PMC9253396

[6] Grembowski D, Paschane D, Diehr P, Katon W, Martin D, Patrick DL. Managed care, physician job satisfaction, and the quality of primary care. J Gen Intern Med. 2005;20(3):271–27. 15836532 10.1111/j.1525-1497.2005.32127.x PMC1490070

[7] DiMatteo MR, Sherbourne CD, Hays RD, et al. Physicians’ characteristics influence patients’ adherence to medical treatment: results from the medical outcomes study. Health Psychol. 1993;12(2):93–102. 8500445 10.1037/0278-6133.12.2.93

[8] Fereidouni A, Teymoori E, Maleki Z, Ghanavati M, Vizeshfar F. Relationships between job satisfaction of operating room nurses and hospital’s compliance with protective guidelines during the Covid-19 pandemic: a cross-sectional study, Iran. J Perianesth Nurs. 2023;38(1):51–7. 35752524 10.1016/j.jopan.2022.03.007 PMC9058135

[9] Zhang T, Feng J, Jiang H, Shen X, Pu B, Gan Y. Association of professional identity, job satisfaction and burnout with turnover intention among general practitioners in China: evidence from a national survey. BMC Health Serv Res. 2021;21(1):382. 33902579 10.1186/s12913-021-06322-6 PMC8074426

[10] Treusch Y, Möckel L, Kohlstedt K. Working conditions, authorizations, mental health, and job satisfaction of physician assistants in Germany. Front Public Health. 2023;11:1082463. 36908456 10.3389/fpubh.2023.1082463 PMC9998044

[11] Malhotra J, Wong E, Thind A. Canadian family physician job satisfaction - is it changing in an evolving practice environment? An analysis of the 2013 national physician survey database. BMC Fam Pract. 2018;19(1):100. 29935531 10.1186/s12875-018-0786-6 PMC6015660

[12] Chen J, Wang Y, Du W, Liu S, Xiao Z, Wu Y. Analysis on the relationship between effort-reward imbalance and job satisfaction among family doctors in China: a cross-sectional study. BMC Health Serv Res. 2022;22(1):992. 35922789 10.1186/s12913-022-08377-5 PMC9351256

[13] Pan B, Shen X, Liu L, Yang Y, Wang L. Factors associated with job satisfaction among university teachers in Northeastern region of China: a cross-sectional study. Int J Environ Res Public Health. 2015;12(10):12761–75. 26473906 10.3390/ijerph121012761 PMC4626998

[14] Shang Guan CY, Li Y, Ma HL. The mediating role of psychological capital on the association between occupational stress and job satisfaction among Township cadres in a specific province of China: a cross-sectional study. Int J Environ Res Public Health. 2017;14(9):972. 28846644 10.3390/ijerph14090972 PMC5615509

[15] Krug EG, Mercy JA, Dahlberg LL, Zwi AB. The world report on violence and health. Lancet. 2002;360(9339):1083–8. 12384003 10.1016/S0140-6736(02)11133-0

[16] Zhang X, Li Y, Yang C, Jiang G. Trends in workplace violence involving health care professionals in China from 2000 to 2020: a review. Med Sci Monit. 2021;27:e928393. 33417590 10.12659/MSM.928393 PMC7802374

[17] Sui G, Liu G, Jia L, Wang L, Yang G. Associations of workplace violence and psychological capital with depressive symptoms and burn-out among doctors in Liaoning, China: a cross-sectional study. BMJ Open. 2019;9(5):e024186. 31129572 10.1136/bmjopen-2018-024186 PMC6538207

[18] CMDA. White Paper on Chinese Physician Practice. http://www.cmda.net/rdxw2/11526.jhtml. Accessed 14 April 2022.

[19] Sun T, Gao L, Li F, et al. Workplace violence, psychological stress, sleep quality and subjective health in Chinese doctors: a large cross-sectional study. BMJ Open. 2017;7(12):e017182. 29222134 10.1136/bmjopen-2017-017182 PMC5728267

[20] Hockey GR. Compensatory control in the regulation of human performance under stress and high workload; a cognitive-energetical framework. Biol Psychol. 1997;45(1–3):73–93. 9083645 10.1016/s0301-0511(96)05223-4

[21] Freudenberger HJ. Staff burn-out. J Soc Issues. 1974(Winter).

[22] Weiss HM, Cropanzano R. Affective events theory: a theoretical discussion of the structure, causes and consequences of affective experiences at work. Res Organ Behav. 1996;18:1–74.

[23] Li N, Zhang L, Xiao G, Chen J, Lu Q. The relationship between workplace violence, job satisfaction and turnover intention in emergency nurses. Int Emerg Nurs. 2019;45:50–5. 30797732 10.1016/j.ienj.2019.02.001

[24] Schlak A, Poghosyan L, Rosa WE, et al. The impact of primary care practice structural capabilities on nurse practitioner burnout, job satisfaction, and intent to leave. Med Care. 2023;61(12):882–9. 37815323 10.1097/MLR.0000000000001931 PMC10695280

[25] Hamdan M, Hamra AA. Burnout among workers in emergency departments in Palestinian hospitals: prevalence and associated factors. BMC Health Serv Res. 2017;17(1):407. 28619081 10.1186/s12913-017-2356-3 PMC5472878

[26] Daemmrich A. The political economy of healthcare reform in china: negotiating public and private. Springerplus. 2013;2:448. 24052932 10.1186/2193-1801-2-448 PMC3776089

[27] Salanova M, Agut S, Peiró JM. Linking organizational resources and work engagement to employee performance and customer loyalty: the mediation of service climate. J Appl Psychol. 2005;90(6):1217–27. 16316275 10.1037/0021-9010.90.6.1217

[28] Schneider B, González-Romá V, Ostroff C, West MA. Organizational climate and culture: reflections on the history of the constructs in the journal of applied psychology. J Appl Psychol. 2017;102(3):468–82. 28125256 10.1037/apl0000090

[29] Olson L. Ethical climate in health care organizations. Int Nurs Rev. 1995;42(3):85–90. 7649724

[30] Zohar D, Livne Y, Tenne-Gazit O, Admi H, Donchin Y. Healthcare climate: a framework for measuring and improving patient safety. Crit Care Med. 2007;35(5):1312–7. 17414090 10.1097/01.CCM.0000262404.10203.C9

[31] Viđak M, Tomić V, Buljan I, Tokalić R, Marušić A. Perception of organizational climate by university staff and students in medicine and humanities: a qualitative study. Acc Res. 2024;31(7):847–73. 10.1080/08989621.2023.2173586 36710428

[32] Xia W, Fan Y, Bai J, Zhang Q, Wen Y. The relationship between organizational climate and job satisfaction of kindergarten teachers: a chain mediation model of occupational stress and emotional labor. Front Psychol. 2024;15:1373892. 38863665 10.3389/fpsyg.2024.1373892 PMC11165699

[33] Caricati L, Sala RL, Marletta G, et al. Work climate, work values and professional commitment as predictors of job satisfaction in nurses. J Nurs Manag. 2014;22(8):984–94. 23890046 10.1111/jonm.12079

[34] Bronkhorst B, Tummers L, Steijn B, Vijverberg D. Organizational climate and employee mental health outcomes: a systematic review of studies in health care organizations. Health Care Manage Rev. 2015;40(3):254–71. 24901297 10.1097/HMR.0000000000000026

[35] Puhanić P, Erić S, Talapko J, Škrlec I. Job satisfaction and burnout in Croatian physiotherapists. Healthc (Basel). 2022;10(5):905. 10.3390/healthcare10050905 PMC9140399 35628042

[36] Chen X, Ran L, Zhang Y, et al. Moderating role of job satisfaction on turnover intention and burnout among workers in primary care institutions: a cross-sectional study. BMC Public Health. 2019;19(1):1526. 31727027 10.1186/s12889-019-7894-7 PMC6857324

[37] Peršolja M, Žvanut B, Rot Š, Markič M. Assessment of management styles among top nursing leaders in Slovenian primary health centers: a cross-sectional analysis. Leadersh Health Serv (Bradf Engl). 2024;ahead–of–print(ahead–of–print):157–68. 38390728 10.1108/LHS-10-2023-0083 PMC11348956

[38] Avolio BJ, Walumbwa FO, Weber TJ. Leadership: current theories, research, and future directions. Annu Rev Psychol. 2009;60:421–49. 18651820 10.1146/annurev.psych.60.110707.163621

[39] Gabele D, Cartwright T, Christen F, et al. Authentic leadership: pearls of wisdom. AACN Adv Crit Care. 2023;34(1):59–62. 36877646 10.4037/aacnacc2023422

[40] Krompa GM, O’Mahony E, Tan J, Mulligan O, Adamis D. The effectiveness of community mental health teams in relation to team cohesion, authentic leadership and size of the team: a study in the North West of Ireland. Community Ment Health J. 2022;58(7):1393–402. 35122580 10.1007/s10597-022-00951-9

[41] Spence Laschinger HK, Wong CA, Grau AL. The influence of authentic leadership on newly graduated nurses’ experiences of workplace bullying, burnout and retention outcomes: a cross-sectional study. Int J Nurs Stud. 2012;49(10):1266–76. 22727121 10.1016/j.ijnurstu.2012.05.012

[42] Iqbal S, Farid T, Khan MK, Zhang Q, Khattak A, Ma J. Bridging the gap between authentic leadership and employees communal relationships through trust. Int J Environ Res Public Health. 2019;17(1):250. 31905864 10.3390/ijerph17010250 PMC6982109

[43] Avolio BJ, Gardner WL, Walumbwa FO, Luthans F, May DR. Unlocking the mask: a look at the process by which authentic leaders impact follower attitudes and behaviors. Leadersh Q. 2004;15:801–23.

[44] Alilyyani B, Wong CA, Cummings G. Antecedents, mediators, and outcomes of authentic leadership in healthcare: a systematic review. Int J Nurs Stud. 2018;83:34–64. 29684833 10.1016/j.ijnurstu.2018.04.001

[45] Laschinger HK, Finegan J, Wilk P. Context matters: the impact of unit leadership and empowerment on nurses’ organizational commitment. J Nurs Adm. 2009;39(5):228–35. 19423988 10.1097/NNA.0b013e3181a23d2b

[46] Wong CA, Laschinger HK. Authentic leadership, performance, and job satisfaction: the mediating role of empowerment. J Adv Nurs. 2013;69(4):947–59. 22764828 10.1111/j.1365-2648.2012.06089.x

[47] Blake N, Blayney F, Loera T, Rowlett C, Schmidt D. A model of authentic leadership to support a healthy work environment. AACN Adv Crit Care. 2012;23(4):358–61. 23095960 10.1097/NCI.0b013e31826b4d1b

[48] Idris MA, Dollard MF, Yulita. Psychosocial safety climate, emotional demands, burnout, and depression: a longitudinal multilevel study in the Malaysian private sector. J Occup Health Psychol. 2014;19(3):291–302. 24802994 10.1037/a0036599

[49] Ren Y, Song H, Li S, Xiao F. Mediating effects of nursing organizational climate on the relationships between empathy and burnout among clinical nurses. J Adv Nurs. 2020;76(11):3048–58. 32885476 10.1111/jan.14525

[50] Appelbaum NP, Lee N, Amendola M, Dodson K, Kaplan B. Surgical resident burnout and job satisfaction: the role of workplace climate and perceived support. J Surg Res. 2019;234:20–5. 30527474 10.1016/j.jss.2018.08.035

[51] Romero-Carazas R, Almanza-Cabe RB, Valero-Ancco VN, et al. Burnout and physical activity as predictors of job satisfaction among Peruvian nurses: the job demands-resources theory. J Prim Care Community Health. 2024;15:21501319241256265. 38813978 10.1177/21501319241256265 PMC11143850

[52] Wang PX, Wang MZ, He GX, Wang ZM. Study on the relationship between workplace violence and work ability among health care professionals in Shangqiu City. J Hyg Res. 2006;35:472–5. 16986527

[53] Miao Q. Handbook of HRM frontier research and practice scales. Zhejiang: Zhejiang University; 2015.

[54] MacDavitt K, Chou SS, Stone PW. Organizational climate and health care outcomes. Jt Comm J Qual Patient Saf. 2007;33(11 Suppl):45–56. 18173165 10.1016/s1553-7250(07)33112-7

[55] Wang X-y, Lin S, Chen L-y, Bai Y. An empirical investigation on the relationship between organization climate, Tacit knowledge sharing behavior and innovation performance. Soft Sci. 2014;28(05):43–7.

[56] Maslach C, Jackson SE. The measurement of experienced burnout. J Organ Behav. 1981;2(2):99–113.

[57] Li C, Shi K. The infuence of distributive justice and procedural justice on job burnout. Acta Psychol Sin. 2003;35(05):677–84.

[58] Sinica AP. Acta Psychologica Sinica OA Statement. https://journal.psych.ac.cn/xlxb/CN/column/column14.shtml. Accessed 13 April 2025.

[59] TU H-W YANM. The differential effects of job design on knowledge workers and manual workers: a field Quasi-experiment in China. Acta Physiol Sinica. 2011;43(07):810–20.

[60] Zhou M, Wang D, Zhou L, Liu Y, Hu Y. The effect of Work-Family conflict on occupational Well-Being among primary and secondary school teachers: the mediating role of psychological capital. Front Public Health. 2021;9:745118. 34778179 10.3389/fpubh.2021.745118 PMC8581260

[61] Zada S, Khan J, Saeed I, Wu H, Zhang Y, Mohamed A, Shame. Does it fit in the workplace? Examining supervisor negative feedback effect on task performance. Psychol Res Behav Manag. 2022;15:2461–75. 36097600 10.2147/PRBM.S370043 PMC9464096

[62] Naqvi AA, AlShayban DM, Ghori SA, et al. Validation of the general medication adherence scale in Saudi patients with chronic diseases. Front Pharmacol. 2019;10:633. 31231222 10.3389/fphar.2019.00633 PMC6558415

[63] Al Zoubi S, Gharaibeh L, Amaireh EA, et al. Unveiling the factors influencing public knowledge and behaviours towards medication errors in jordan: a cross-sectional study. BMC Health Serv Res. 2024;24(1):798. 38987809 10.1186/s12913-024-11230-6 PMC11238437

[64] Marsh HW, Hau K, Wen Z. Search of golden rules: comment on hypothe sis-Testing approaches to setting cutoff values for fit indexes and dangers in overgeneralizing Hu and bentler’s (1999) findings. Struct Equation Modeling: Multidisciplinary J. 2004;3(11):320–41.

[65] Wu M-L. Structural equation modeling: operation and application of AMOS. Chongqing University Press; 2010.

[66] Byrne BM. Structural equation modeling with EQS and EQS-Windows: basic concepts, applications, and programming. Sage Publications, Inc.; 1994.

[67] Hu H, Peng B, Chen W, Wang H, Yu T. How psychological resilience shapes adolescents’ sports participation: the mediating effect of exercise motivation. Front Psychol. 2025;16:1546754. 40271354 10.3389/fpsyg.2025.1546754 PMC12014653

[68] Hair J, Black W, Babin B, Anderson R. Multivariate data analysis: a global perspective; 2010.

[69] Strauss ME, Smith GT. Construct validity: advances in theory and methodology. Annu Rev Clin Psychol. 2009;5:1–25. 19086835 10.1146/annurev.clinpsy.032408.153639 PMC2739261

[70] Shrout PE, Bolger N. Mediation in experimental and nonexperimental studies: new procedures and recommendations. Psychol Methods. 2002;7(4):422–45. 12530702

[71] Wu Y, Buljac-Samardzic M, Zhao D, Ahaus CTB. The importance and feasibility of hospital interventions to prevent and manage patient aggression and violence against physicians in china: a Delphi study. Hum Resour Health. 2024;22(1):34. 38802830 10.1186/s12960-024-00914-z PMC11131301

[72] General office of the state council. national performance assessment manual for tertiary public hospitals. (2024 Edition). Accessed 15 Mar 2024. http://www.nhc.gov.cn/yzygj/s3594q/202403/94a97921a9b043e8b8e3315aed9f1627.shtml

[73] Wang QL, Liu P, Zhou TR. Job satisfaction and its influencing factor of general practitioners in primary medical institutions in Yinchuan. Med Philoso. 2022;43(6):69–72.

[74] Wu ZW, Chen Y, Li DS, et al. Canonical correlation analysis on job burnout and organizational support among medical staffs. Chin J Occup Med. 2019;46(3):322–325330.

[75] Qi L, Wei X, Li Y, Liu B, Xu Z. The influence of mistreatment by patients on job satisfaction and turnover intention among Chinese nurses: a three-wave survey. Int J Environ Res Public Health. 2020;17(4):1256. 32075294 10.3390/ijerph17041256 PMC7068529

[76] Brewer KC, Dierkes AM, Norful AA. Organizational trust breaches among nurses and aides: a qualitative study. Nurs Ethics. 2024;31(8):1524–36. 38417902 10.1177/09697330241230520 PMC11349928

[77] Ma Y, Ni X, Shi Y, et al. Epidemic characteristics and related risk factors of occupational exposure for pediatric health care workers in Chinese public hospitals: a cross-sectional study. BMC Public Health. 2019;19(1):1453. 31690294 10.1186/s12889-019-7862-2 PMC6833173

[78] Irving D, Page B, Carthey J, Higham H, Undre S, Vincent C. Adaptive strategies used by surgical teams under pressure: an interview study among senior healthcare professionals in four major hospitals in the United Kingdom. Patient Saf Surg. 2024;18(1):8. 38383433 10.1186/s13037-024-00390-3 PMC10880194

[79] Schneider B, Ehrhart MG, Macey WH. Organizational climate and culture. Annu Rev Psychol. 2013;64:361–88. 22856467 10.1146/annurev-psych-113011-143809

[80] Almutairi M, Timmins F, Wise PY, Stokes D, Alharbi TAF. Authentic leadership-a concept analysis. J Adv Nurs. 2025;81(4):1775–93. 39425899 10.1111/jan.16496 PMC11896828

[81] Raso R, Fitzpatrick JJ, Masick K. Clinical nurses’ perceptions of authentic nurse leadership and healthy work environment. J Nurs Adm. 2020;50(9):489–94. 32826518 10.1097/NNA.0000000000000921

[82] Lindsay SL, Mathieson KM. Authentic leadership: does it relate to job satisfaction and engagement? Nurs Manage. 2022;53(6):24–30. 35575268 10.1097/01.NUMA.0000831416.21965.e2

[83] Alboroto R, Garza T, McNaughtan J. Enhancing caseworker job satisfaction through empowerment: an innovative tool for employee retention. Child Maltreat. 2025;30(3):553–64. 39752415 10.1177/10775595241313134

[84] Taskiran Eskici G, Uysal Kasap E, Gumus E. Relationships between leadership behaviour of nurse managers and nurses’ levels of job satisfaction and compassion fatigue during the COVID-19 pandemic. Nurs Open. 2023;10(7):4548–59. 36879354 10.1002/nop2.1701 PMC10277410

